# Perspectives on Rising Societal Crime on Workplace Productivity in a Small Island Developing State

**DOI:** 10.3390/ijerph22121858

**Published:** 2025-12-12

**Authors:** Adeoye Adenekan, Marsha Ivey, Srikanta Banerjee

**Affiliations:** 1 Department of Public Health and Primary Care, Faculty of Medical Sciences, University of the West Indies, St. Augustine, Trinidad and Tobago; marsha.ivey@uwi.edu; 2 Core Faculty, School of Health Sciences and Public Policy, Walden University, Minneapolis, MN 55401, USA; srikanta.banerjee2@mail.waldenu.edu

**Keywords:** impact of societal crime, phenomenological qualitative research, work productivity, university, Trinidad

## Abstract

**Objectives:** The crime rate in Trinidad and Tobago has increased over the last few years. It is important to understand the impact of rising societal crime on university workplace productivity in order to make meaningful recommendations to mitigate the negative effects of crime. **Methods:** We conducted semi-structured interviews online via Zoom and face-to-face with both academic and non-academic staff from a university located in Trinidad and Tobago in April 2025. We employed purposive sampling and topics explored included participants’ views on crime, the effect of crime on workplace productivity, the effect of crime on workplace concentration, the effect of crime on participants’ mental health, concerns about safety at the workplace, and desired changes or suggestions to ensure improved safety at the workplace. Data were manually analyzed, and we employed thematic analysis to understand the participants’ data. **Results:** Analysis included data from 10 participants. Participants represented both academic and non-academic staff, with varied ethnic backgrounds, age range, and were both from Mount Hope and the main campus. Seven of the participants believed that their work productivity had been negatively affected by the crime situation. All the participants agreed that the crime situation was out of control; two of the participants claimed to have been victims of crime. Five of the participants believed they had experienced depressive symptoms, while six participants claimed to have experienced poor concentration on the job. Five participants expressed genuine concerns that something terrible could happen to them within their workplace premises. In order to improve security at the workplace, seven of the participants suggested the employment of more security personnel, while six participants highlighted the need for more surveillance and closed-circuit television (CCTV) cameras. Participants identified four major categories or themes: views on crime and its effects on individuals; effects of crime on workplace productivity; effects of crime on mental well-being; and suggestions and opportunities to improve security at the workplace. **Conclusions:** From this study, it can be inferred that the majority of the participants were negatively affected by the climate of crime in the country. A comprehensive risk assessment would identify potential risks and vulnerabilities faced by staff, while enhanced surveillance measures and the promotion of the Employee Assistance Program (EAP) can support those impacted. Staff should also be trained to respond effectively to potential threats.

## 1. Introduction

There has been a sudden increase in societal crime and gang-related violence in several communities in Trinidad and Tobago over the last five years [1]. This trend in the level of crime is predicted to continue in the coming years unless there are significant policy changes to address this societal problem [1]. Presently, the level of homicides in Trinidad and Tobago has reached an alarming level of 413 cases as of 26 August 2024, surpassing the same statistics from the same period over the last five years [1]. Furthermore, with a homicide rate of 26 per 100,000 people, Trinidad and Tobago not only exceeded the homicide rates in Colombia and Mexico in 2023, but the level will likely surpass the record 605 murders reported in the country in 2022 [1].

Social and economic development in many Latin American and Caribbean countries (LAC) is being stymied by crime and violence [2]. Crime continues to be a significant burden on societies in these countries and on their economic development, which further limits growth, drives inequality and results in unnecessary expenditure by both private and public entities to combat crime [2]. Public expenditure on crime indirectly impacts or reduces funding on other critical sectors of the economy, such as education, social assistance, health, infrastructural development and research [2]. More importantly, crime and violence result in loss of human capital [2]; it was estimated in 2022 that in many of the LAC countries, security expenses by private businesses accounted for 47% of the total cost of crime, state spending on crime prevention represented 31%, and the loss of human capital made up 22% [2].

There is a growing body of research evidence that links residential or societal neighborhood crime to mental health disorders. Important neighborhood stressors such as local crime and violence may be associated with people’s mental health through different pathways [3]. Similarly, crime and violence in society are major public health concerns and are included in the Sustainable Development Goals [3].

In Trinidad and Tobago, mental, neurological, substance use disorders and suicide (MNSS) account for 16% of all disability-adjusted life years (DALYs) and 31% of all years lived with disability (YLDs) [4]. An individual’s mental health status across life course is often affected by some broader social determinants of health, such as the prevailing structural components of the individual’s immediate environment [5]. These structural components may account for the differences in mental health outcomes across populations [5]. Some of these structural components include, but are not limited to, factors such as social support and the neighborhood’s social and physical conditions in which people live [5]. Elderly people who have been victims of violence have an increased risk of poor health outcomes [6]. Furthermore, the incidence of mental health problems such as major depression and eating disorders, as well as physical injuries, has been found to be more prevalent among women subjected to partner violence and a variety of societal violent experiences [6]. Increased stress associated with depressive illness, fear, and anxiety disorders is likely to be experienced by older adults who have been subjected to abuse [6]. Similarly, it has been documented that the fear associated with neighborhood crime promotes social instability and is detrimental to one’s self-rated mental and physical well-being [6]. From the foregoing, it can be expected that one may experience poor mental health outcomes in an environment or neighborhood where violence and crime thrive. However, what is not known is whether people’s productivity at their places of work can be negatively affected by living in such a crime and violence-riddled environment.

## 2. Methods

### 2.1. Setting, Participants, Recruitment, and Sample Size

We conducted a phenomenological qualitative study [7] in April 2025 with ten semi-structured interviews using either face-to-face or online via Zoom. A phenomenological design was the most suitable method, as the design allows researchers to understand participants’ lived experiences and perspectives regarding a specific phenomenon [7]. Participants were recruited from the staff of a tertiary academic institution located in Trinidad and Tobago. The participants all resided in Trinidad. Purposive sampling [7] was used to identify potential participants from both the academic and non-academic categories, the two broad categories of staff at the university, and ethnic backgrounds (East Indian, African and Mixed descent), who were adequately represented in our study sample. East Indian, African, and Mixed descent are the three main ethnic groups in Trinidad and Tobago. Identified staff were emailed an invitation with the participant’s information sheet, which outlined the purpose of the study and the inclusion criteria (persons ≥ 18 years, employed with the institution, resident in Trinidad and Tobago, conversant in English and able to provide signed consent) and the exclusion criteria. Those who agreed to participate were asked to reply to the email message, after which they were sent a consent form to obtain their written informed consent.

Participants were offered the option of either face-to-face or online semi-structured interviews at a mutually agreed date and time. We targeted five to 25 participants as recommended for phenomenological studies [8], but at least six participants are also sufficient [9]. Hence, 10 participants were recruited for this phenomenological qualitative study.

The study was approved by the University of the West Indies Campus Research Ethics Committee (CREC-SA.3101/02/2025).

### 2.2. Data Collection and Analysis

Semi-structured interviews, which lasted 30 min, were conducted using 10 guiding questions with the participants on different days and times in April 2025 (see Table 1). The questions covered: demographics, views on crime, workplace productivity, mental health issues, and suggestions for improved security at the workplace. Workplace productivity was defined as the ability of an individual, team, or organization to work efficiently in a timely manner to maximize output and achieve their aims and objectives [10]. Participation was voluntary, and participants could decline to answer any question or leave at any time. For the face-to-face interviews, participants’ responses were audio-recorded with the voice recorder app of the researcher’s laptop while for online interviews, a recording and Zoom summary were generated. The anonymization process was ensured, where participants were assigned a unique identification number. This process ensured that the participants’ responses cannot be linked to them. Confidentiality was also ensured by not collecting any identifiable information from the participants before and during the data collection process.

The data were transcribed verbatim [11]. We ensured the reliability of the analytical procedure by being objective and ensuring that our personal biases as researchers did not affect the data analysis [12]. One team member reviewed transcripts alongside audio recordings and summaries from Zoom to ensure accuracy. We ensured study validity by employing participant validation [12]. Participants were emailed their transcripts and were asked to confirm that the contents of the transcripts were consistent with the interviews they gave. Thematic analysis [13] was used to identify codes and group them into major and minor themes [13]. Participants’ demographic data were analyzed with descriptive statistics.

Two researchers carried out thematic analysis of the semi-structured interview data, independently generating data codes based on the semi-structured interview guide. These two reviewers then met to compare analyses and come to a consensus about major codes or themes and minor categories [13]. All the data were then reorganized based upon this agreed categorization [11]. Data from the minor codes or categories were merged to form the major themes to help structure the subsequent result reporting.

## 3. Results

### 3.1. Participant Characteristics

Participant demographic characteristics are shown in Table 2. The majority of the participants were females, with participants’ ages spanning over a 20-year range. All participants claimed to be of middle socioeconomic status, with most participants being of Afro-Caribbean descent and having post-graduate level education.

Participants’ responses were grouped into four major themes or categories: views on crime and its effects on individuals, effects of crime on workplace productivity, effects of crime on mental well-being, and suggestions and opportunities to improve security at the workplace (See Table 3 for additional detail).

### 3.2. Views on Crime and Its Perceived Effect on Individuals

Across all interviews, the same view was expressed that the “state of crime in the country was out of control”—P2. Additionally, participants highlighted different ways in which the state of crime had “disrupted their lives and daily routine”—P1.

Participant 10 described the crime situation as having *“affected me in so many ways… we have had to take extreme precautions to secure and modify our home. For example, we had to raise the walls, reinforce the windows and doors, install an automatic gate and a security alarm system… Of course, all these came at a great cost to us.”*

Not only has the crime situation affected the finances, safety, and security of participants at their homes, but it has also affected their daily movements and social engagements. One participant lamented the fear of being out on the road, even when travelling to work because:

*“The crime situation in the country is so horrible that it actually came across my mind several times to do other things… such as farming to sustain myself financially, so I wouldn’t have to be travelling to work on the road which to me is not safe nowadays…”* Participant 7. Others stated, *“You are scared to be out there because you cannot tell what next is going to happen.”*- Participant 4. *“It has become uncomfortable travelling at certain hours, typically after dark…you try not to be out around 7 p.m. or 8 p.m.”* Participant 5.

The issue of gang wars was another negative effect of crime highlighted by participants. For example, Participant 8 posited his view that *“raging gang wars and the emergence of new gangs…coupled with the lack of effective measures from different governments and law enforcement agencies have significantly contributed to the terrible state of crime in the country.”* In her view, Participant 9 highlighted *“crime has drastically increased in the country, with the associated gang wars and patterns of crime that we had never seen before in this country.”*

### 3.3. Effects of Crime on Workplace Productivity

Several participants described how they believed the crime environment had affected their workplace productivity. Participant 1 reflected that, *“When I was a graduate student, I would come to the lab at any hour of the day… so that is restricted. When I design studies, I must think about that…I must think about my students.”* Additional stress and burden were highlighted as factors that have impacted workplace productivity. One participant believed that the crime environment *“… in the country has impacted my work productivity because more time must be spent on investigating substantially increased numbers of crime related cases or complaints at the workplace…this situation has put an extra burden on other workers who work shift duty as they too must stay behind after their regular hours… to investigate numerous cases and incidents.”—P*2

However, not all reactions to the crime environment experienced by the participants were negative. Some participants claimed their work productivity had not been impacted by the state of crime. For example, for Participant 4 *“The crime situation has not impacted my work productivity… but I have had concerns at times whilst at work which impacted on my concentration”,* while Participant 6 shared a positive perspective on work productivity, because *“The crime situation in the country has not impacted on my work productivity…I feel more secure at work with my colleagues and security personnel around.”*

### 3.4. Effects of Crime on Mental Well-Being

It is common knowledge that being in a good state of mental well-being augurs well for one’s health. Several participants highlighted how the climate of crime had affected their mental well-being. As Participant 1 explained that *“while she had not particularly felt depressed about the crime situation, there was a particular occasion when we had invited a team of people from abroad to come to my department…When they read in the news about the state of crime in the country, they declined the invitation from my department due to safety concerns…To me, that was depressing.”* Furthermore, another participant who claimed to have been a victim of a home invasion, offered that she had: *“…had panic situations in recent past, where my security alarm at home would sound off…I would leave work and rush back home…I have not felt depressed, but I have been very angry because I was invaded, and I feel I could still be invaded again.”*—P9. Some participants reported having been traumatized by crimes committed against their colleagues. For example, Participant 5 shared that *“…a situation occurred a couple of years ago…One of my coworkers was declared missing, only to be found dead a few days later…I was traumatized for days that followed.”*

### 3.5. Suggestions and Opportunities to Improve Security at the Workplace

Participants offered suggestions and ideas that they believed could improve security at their workplace. Several participants were quite optimistic that the implementation of these measures would improve the security situation. Participant 10 suggested, *“…the university should employ more security personnel…There should be regular checks of people entering the campus and an enhanced implementation of CCTV cameras…Entry points into the main campus should be marked and closed after regular office hours…Lightening on the campus can be improved, especially in the evening, by leaving the lights on till 9 p.m. This will enhance the safety of those moving through the campus after classes end at 8 p.m.”*

Participant 7 took issue with the university’s policy of hiring contracted security firms on the campus, stating that, *“…the campus management team tends to be reactive in their approach to security issues on campus…Having a contracted security team on the university campus is a threat for me…Contracted security personnel might not have that intimate relationship with workers and students alike, which can enhance security on campus.” However, not* all the participants agreed on the need to employ more security personnel. Participant 9 believes that *“The university does not necessarily have to employ more security personnel…They need to be strategically deployed within the workplace premises…There is a need for an alarm system in all offices that can be easily activated in emergency situations.”* In contrast, some participants were of the view that workers also had important roles to play in ensuring safety and reducing crime in the workplace. As Participant 2 opined, *“Workers, as well as students, should be more responsible on the workplace premises…They should not make themselves easy targets for criminals…They should desist from leaving valuable items exposed in their cars…Students should properly secure their items such as laptops while on the university premises.”*

## 4. Discussion

This exploratory study provides important context about the challenges that workers face in an environment or society where crime thrives, across four key areas: views on crime and its effects on individuals; effects of crime on workplace productivity; effects of crime on mental well-being; and suggestions and opportunities to improve security at the workplace. The first-hand accounts of workers reported herein, coupled with available statistical data and the extant literature on the impact of societal crime on social and mental well-being [14,15], underscore the public health significance of the workers’ experiences and perspectives, especially as it relates to their mental well-being and workplace productivity.

There is the need for a more comprehensive approach to addressing workers’ plight and challenges at the workplace, as it relates to societal crime: addressing the social determinants of crime at the societal level, novel strategies to combat crime within society and the country in general, improved and enhanced security initiatives at the workplace, and crime prevention through environmental design [CPTED] [16]. These firsthand, experiential accounts create an opportunity for wider population-based studies on the effects of crime on workplace productivity, intervention testing, and policy development.

Several participants’ perspectives align with broader awareness of the effects of societal crime on individuals and communities [17]. Such individuals who are constantly exposed to the negative effects of high levels of societal crime face a greater risk of mental health problems such as depression and post-traumatic stress disorder (PTSD) [18]. Crime impacts public health outcomes, and living in an area associated with high levels of violence can lead to high levels of stress, which in turn seems to increase the likelihood of the development of mental health problems [18]. Very few people are victims of violent crime, but the fear and distress associated with violent crime affect the quality of life [19]. The fear associated with violence disproportionately affects the vulnerable—the poor, women and minority groups [19].

Addressing these negative effects of crime on workers’ well-being is vital to any systematic efforts aimed at improving workplace productivity and enabling workers to contribute meaningfully to national development. The goal of the national mental health policy is to promote the mental well-being of all people of Trinidad and Tobago, while encouraging people to thrive and make meaningful contributions to their communities [20]. This goal cannot be achieved in an environment where crime and violence thrive.

Solutions are needed to address the negative effects of crime on productivity in the workplace and the impact of crime on workers’ well-being. In a study, a positive work environment improved employee performance [21]; similarly, a positive work environment also significantly improved the employee commitment level and achievement-striving ability [21]. From the foregoing, measures such as access control to buildings, where key cards or biometric systems are employed to limit entry to authorized personnel, and surveillance systems where cameras are installed in strategic locations, can deter criminal activity, monitor incidents and improve the overall work environment. Adequate lighting in and around the workplace to enhance visibility and deter crime, along with regular maintenance of the premises to eliminate hiding spots and hazards, can also enhance the workplace environment and improve workplace productivity and employee well-being.

Fostering a positive workplace culture is another important strategy to mitigate the impact of crime on workplace productivity. This can be achieved by encouraging teamwork and communication among employees to create a supportive environment. Furthermore, employee engagement through the involvement of employees in safety initiatives to increase their commitment to maintaining a secure workplace can ultimately lead to an enhancement in the workplace environment. A comprehensive risk assessment to identify potential risks and vulnerabilities faced by staff would be a good starting point to promote employees’ mental well-being in the workplace.

A positive organizational culture that supports workers’ mental health, by incorporating mental health into the organization’s human capital strategy, governance and leadership, can lead to an overall enhancement of employees’ well-being [22]. Furthermore, the provision and utilization of stress management practices that provide employee resources, address organizational issues that cause stress, and reduce physical and psychosocial stressors in the work environment can enhance employees’ mental well-being [22]. Employee Assistance Programs (EAPs) are confidential services designed to support employees by providing them with resources like counselling, financial advice, and family support to help them navigate personal and work-related challenges. Evidence also supports that mental health training and a robust EAP accessible to employees can improve the mental well-being and health outcomes of a diverse workforce population [22].

Public health relevance:
  - This study is relevant in that it highlights the challenges faced by workers in societies with high levels of crime rate;
  - The study is also relevant in that it highlights the health burden associated with working in an environment where crime thrives.

Public health significance:
  - This study highlighted workers’ experiences and perspectives on their well-being as it relates to workplace productivity;
  - This study highlights the need for a more comprehensive approach to addressing workers’ plight and challenges at the workplace, as it relates to societal crime.

Public health implications:
  - High levels of societal crime will prevent people from making meaningful contributions to their communities;
  - The implications could also mean higher rates of mental conditions such as depression, post-traumatic stress disorder, or even premature death.

## 5. Limitations

This exploratory study explored the perspectives and experiences of some individual workers. This focus on individual experiences of workers might have overlooked broader social, cultural, or systemic factors that influenced those experiences. Additionally, given the study’s regional focus on workers at a particular institution, the views and experiences expressed by the study participants may not be generalizable to wider workforce populations in the country.

## 6. Conclusions

The key findings presented here highlight the impact of the climate of crime on workers’ productivity and well-being in an institution and identify the opportunity to institute measures to mitigate the negative effects of crime. Careful attention should be directed to promoting a more secure workplace environment and measures designed to enhance workers’ well-being. Policymakers and institutional authorities should consider the experiences identified here to design and test novel interventions to improve workers’ productivity and promote their mental well-being.

## Figures and Tables

**Table 1:** Semi-structured interview guide sample, Trinidad 2025.

Semi-Structured Interview Guide Sample
**Participant’s Unique Research Number:**
**Demographic Information:** A. Age B. Gender C. Educational Status D. Socioeconomic Status
1. Can you tell me about your views on crime in the country (Trinidad and Tobago) within the last couple of years?
2. In what ways has the crime situation affected you?
3. Do you work alone on your job, or do you work as a member of a team?
4. Work productivity is defined as the ability of an individual, team, or organization to work efficiently in a timely manner to maximize output and achieve their aims and objectives. How has the crime situation in the country affected your work productivity?
5. Have you ever decided at any point within the last couple of years not to come to work or apply for sick leave, because of the crime rate in the country? If so, can you briefly explain?
6. Was there any period or time within the last few years when you felt you couldn’t concentrate at your workplace because of concerns about the crime rate in the country? If so, can you briefly explain?
7. Was there any period or time within the last few years when you panicked and/or felt depressed at your workplace because of concerns about the crime rate in the country? If so, can you briefly explain?
8. Have you ever had any concerns within the last few years that something terrible could happen to you, such as being attacked, within the premises of your workplace, as a result of the crime situation in the country? If so, can you briefly explain?
9. Do you think there are any additional measures that can be taken by your employers to ensure safety and address the concerns of workers like you as it relates to the crime situation, especially when at work? If so, can you briefly explain?
10. Are there any other issues you would like to discuss with me, as they relate to this research study?

**Table 2:** Demographics of Participants (*n* = 10) Reporting on Rising Societal Crime on University Workplace Productivity, Trinidad.

Demographics Characteristics	*n* (%)
Age (years):	
Range	38 to 58
Average	47.2
Gender:	
Male	4 (40%)
Female	6 (60%)
Ethnicity:	
Afro-Caribbean	4 (40%)
Indo-Trinidadian	3 (30%)
Mixed	3 (30%)
Education:	
Post-graduate	4 (40%)
Graduate	3 (30%)
High school	3 (30%)
Socioeconomic status:	
Low	0 (0%)
Middle	10 (100%)
High	0 (0%)
Employment Category:	
Academic	5 (50%)
Non-academic	5 (50%)

**Table 3:** Themes and Participant Examples of Societal Crime on University Workplace Productivity, Trinidad 2025.

Theme	Description	Concepts	Example of Participant Quote
Views on crime and its effect on individuals	Ways in which workers are affected by crime	Fear of being on the road Fear of being a victim Heightened Fear Increased caution	“The situation has become increasingly scary to the point where one feels restricted whether socially or work related.”—P7 “Crime has drastically increased... We are witnessing patterns of crime never seen before.”—P9
Effects of crime on workplace productivity	Ways in which crime has affected workers’ output	Safety at workplace Increased burden on workers Distraction on the job	“I try to stay on campus and get some work done after working hours…Because of the crime situation now, I’m unable to do that any longer.”—P10 “This situation has put an extra burden on other workers who work shift duty… They too have to stay behind after their regular hours to investigate numerous cases.”—P2 “Sometimes at work, you have to call home to check on their safety…You are worried about them and because of this, it affects your productivity.”—P3
Effects of crime on mental well-being	Ways in which crime has affected workers’ mental health	Feelings of depression Poor concentration Panic situations	“I had felt depressed about the crime situation, in terms of the murders and the realization that the measures put in place to fight crime are not effective.”—P8 “I have been a victim of crime… This has impacted my work and emotional well-being.”—P6 “There were a couple of occasions when I panicked at work…If I heard of a crime-related news, I would worry about my loved ones.”—P10
Suggestions and opportunities to improve security at the workplace	Ways to improve security at the workplace	More surveillance (CCTV) cameras Stop and interview members of the public Employment of more security personnel	“To improve security at the workplace, more CCTV cameras should be installed, as well as more security patrols.”—P1 “Such members of the public could be stopped and interviewed…The members of the public should be made to understand that they cannot just walk into the university premises as they wished.”—P3 “Inadequate security staff is giving the intruders the opportunity to invade the university farms and steal from the university’s premises.”—P2

## Data Availability

Due to ethical and privacy reasons, data for this research study are not publicly available. However, the corresponding author can be contacted for any requests regarding the data for this study.
